# Transcriptome Analysis Reveals Co-Expression Regulation of Sugar Transport and Signaling Networks in Initiating Stolon-to-Tuber Transition in Potato

**DOI:** 10.3390/ijms26115278

**Published:** 2025-05-30

**Authors:** Jun Hu, Jinxue Hu, Shaoguang Duan, Congchao Xiang, Yanfeng Duan, Shuqing Zhang, Guangcun Li

**Affiliations:** 1 State Key Laboratory of Vegetable Biobreeding, Key Laboratory of Biology and Genetic Improvement of Tuber and Root Crops, Ministry of Agriculture and Rural Affairs of the People’s Republic of China, Institute of Vegetables and Flowers, Chinese Academy of Agricultural Sciences, Beijing 100081, China; hujun@caas.cn (J.H.);; 2 Shijiazhuang Academy of Agriculture and Forestry Sciences, Shijiazhuang 050041, China; hujinxue93@163.com (J.H.);

**Keywords:** potato, transcriptome analysis, tuber initiation, maturity, regulatory network

## Abstract

The network regulatory mechanism governing the dynamics of plant maturity and tuber development in potatoes (*Solanum tuberosum* L.) has remained a major focus in potato molecular biology research. In this study, three potato cultivars with different maturity periods (‘Shishu 2’, ‘Zhongshu 3’, and ‘Zhongshu 49’) were examined. RNA sequencing was performed on samples from five tissues, including the leaves, stems, stolon (T0), sub-apical swellings (T1), and initiation stage (T2), to reveal the co-expression regulatory network involved in leaf, stem, and tuber development. *StSWEET11* and *StSP6A* were significantly upregulated in the early-maturing cultivar ‘Shishu 2’. Differentially expressed genes were classified into 18 modules (ME) using weighted gene co-expression network analysis (WGCNA). Among these, ME1, ME3, and ME13 showed significant positive correlations with leaf tissue, ME2, ME4, and ME15 with stem tissue, and ME7, ME8, and ME14 with T1 and T2 tissues. *StSP5G* was identified as the core hub gene of ME4. Genes such as *StCOL1*, *StSWEET11*, and *StSP6A* exhibited significant co-expression in leaf-related modules. *StGIGANTEA* and *StGIGANTEA-like* played important regulatory roles in linking the expression networks of stems and tubers. Metabolism-related genes, including *StSUSⅠc*/*StSuSy4* and *StDPE1*, were also found to be crucial in mediating interactions between leaf and tuber tissues. Therefore, this study provides new insights into the regulatory network governing tuberous signal transmission from leaves and stems to tubers.

## 1. Introduction

The regulatory network mechanism underlying potato maturity and the initiation and development of tubers has long been a focal point of research in potato molecular biology [1,2,3]. Potato cultivars with distinct maturation periods exhibit pronounced differences in growth cycles, yields, and quality. These disparities directly influence varietal adaptability to diverse environments and ultimately affect tuber yield, significantly impacting economic returns in agricultural production [4]. The formation and development of potato tubers is a complex process, spanning a series of stages from stolon formation, elongation, sub-apical swelling, and initial tuber formation to tuber expansion and maturity. This process is regulated by a combination of environmental and metabolic factors, including photoperiod, temperature, water and nutrient availability, and hormones [2,3]. Therefore, the in-depth exploration of the molecular regulatory mechanism of potato plant maturity and tuber development is of theoretical and practical significance for expanding the regional adaptability of potatoes and enhancing their tuber yield potential.

The initial formation of tubers is closely regulated by the photoperiod [5]. Previous studies have shown that the tuber-inducing signal molecule SELF PRUNING 6A (SP6A) is produced in leaves under short-day conditions and transported to the stolon through vascular bundles, stimulating tuber formation [6]. *SP6A* expression is negatively regulated by the CONSTANS (CO) protein [7]. Under long-day conditions, CO transcriptionally activates SP5G, a repressor of *SP6A*, and *SP5G* inhibits SP6A expression. The stability of CO is maintained by PHYB and PHYF proteins [8,9], while its transcription is increased through the proteolytic degradation of the CDF1 protein, mediated by the FLAVIN-BINDING KELCH REPEAT F-BOX PROTEIN 1/GIGANTEA (FKF1/GI) complex [10]. Under short-day conditions, CDF1 avoids degradation through the FKF1/GI complex, thereby inhibiting *CO* expression and indirectly activating SP6A expression [11]. StSP6A, the St14-3-3 protein, and StFDL1 form a tuberigen complex that regulates early tuber development [12,13]. Both *SP6A* and *CDF1* expression are transcriptionally activated by the BEL5 protein, which acts synergistically with POTH1 [14,15].

Plant hormones regulate different stages of tuber formation [16]. Gibberellin (GA), a key hormone in this process, promotes potato stolon elongation [16]. Exogenous GA application enhances stolon elongation but inhibits tuber formation. However, high concentrations of sucrose can counteract GA’s inhibitory effect. Cytokinin [16] and auxin [17] also play important roles in promoting tuber formation by regulating cell division, cell expansion, and storage metabolism during tuberization. Abscisic acid (ABA) is generally considered inhibitory to tuber formation, though under specific conditions, it can enhance the process [18].

As the primary transportable carbohydrate in plant metabolism, sucrose provides energy for tuber growth and acts as a key signaling molecule in tuberization [19,20,21]. In potato stolons, sucrose is exported to the apoplast via Sugars Will Eventually Be Exported Transporter (SWEET) proteins, maintaining an appropriate sucrose concentration in stolon cells [22]. A study showed that the SP6A protein binds to the SWEET protein at the stolon tip, blocking apoplastic sucrose export and increasing intracellular sucrose levels [23]. Sugar transporter *StSWEET1g* affects the apoplasmic sugar ratio and phloem-mobile tuber signals [24]. This triggers cell division and expansion in the sub-apical stolon region, increasing the number of parenchyma cells, which are rich in symplastic connections and facilitating starch storage [23,24]. However, the specific mechanism by which the tuber-forming signal regulatory network is transmitted from leaves to stolons still requires further research.

Transcriptomics technology provides a powerful tool for in-depth research on the molecular mechanisms of potato tuber development [25]. Transcriptome analysis enables a comprehensive understanding of changes in gene expression profiles in potatoes across developmental stages and environmental conditions, revealing key genes and regulatory networks involved in tuber initiation and stolon-to-tuber transition [25,26]. Differences in tuber initiation and stolon development among potato cultivars may be closely linked to gene expression regulation. Therefore, comparative transcriptome studies of cultivars with different maturities can enhance our understanding of the molecular mechanisms governing tuber initiation and stolon development, providing an important theoretical basis for genetic improvement and breeding.

This study aimed to apply transcriptomics technology to systematically analyze gene expression in potato cultivars with different maturity periods during the initial development of leaves, stems, and tubers, as well as stolon-to-tuber transition. By comparing gene expression differences across cultivars, we aimed to identify key genes and regulatory networks involved in tuber initiation and stolon development, thus providing new insights into their regulatory mechanisms.

## 2. Results

### 2.1. Analysis of Transcriptome Sequencing

**Figure 1:**
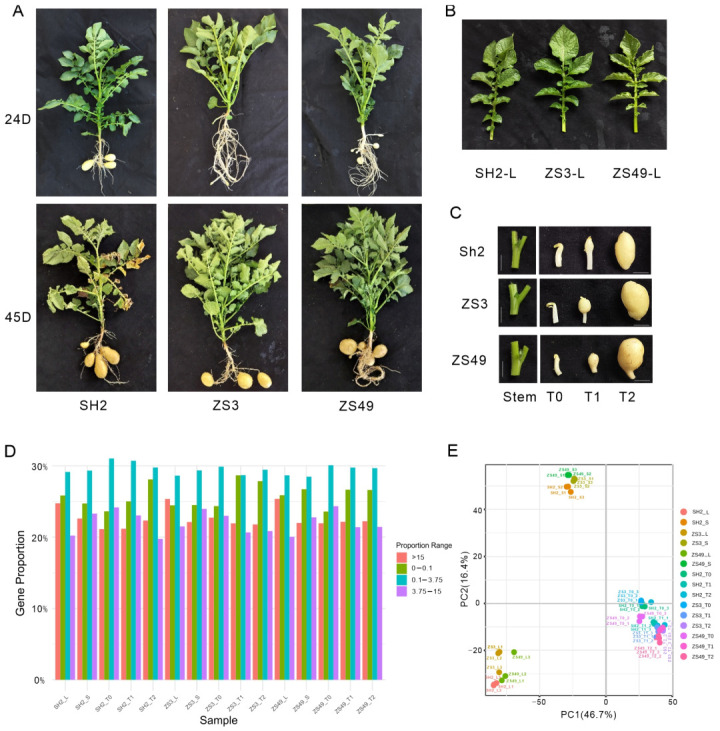
Comparative analysis of morphology and global transcriptome across different tissues of three potato cultivars. (**A**) Developmental comparison of leaves and tubers in three potatoes cultivars at 24 and 45 days after emergence. (**B**–**C**) Tissue samples of leaves, stems, and stolons (T0 stage, where stolons remain unswollen), as well as tubers (T1 and T2 stages, representing the continuous swelling phase) from three potato cultivars, scale bar of 5 mm. (**D**) Global transcriptome expression levels across five tissue types in the three cultivars. (**E**) Principal component analysis of gene expression differences across 45 tissue samples from the three cultivars.

To analyze the regulatory network of potato leaves, stems, and underground stolons, as well as the initial formation and development of tubers, this study selected three potato cultivars with different maturity periods (‘Shishu 2’ (SH2), ‘Zhongshu 3’ (ZS3), and ‘Zhongshu 49’ (ZS49)) and conducted transcriptome sequencing on leaf and stem tissues as well as samples from three developmental stages (T0, T1, and T2) of tuber initiation and formation (Figure 1A,B). This study included three biological replicates, totaling 45 samples. The sequencing data (clean base) per sample ranged from 5.53G to 8.12G, with sequencing quality (Q30) exceeding 95.5%. Among the processed clean reads, 82.3% (ranging from 79.72% to 85.57%) aligned to the reference genome (Supplementary Table S1). A total of 28,653 genes with fragments per kilobase of exon model per million mapped reads (FPKM) greater than 0.1 were detected (Supplementary Table S2), representing 71.3% to 76.4% of all genes. Principal component analysis (PCA) showed that samples from the same tissue type, including leaves, stems, and tubers at different developmental stages (T0, T1, and T2), were clustered together, with gene expression differences among the tissue types being significantly greater than those among the cultivars (Figure 1E). Additionally, an expression correlation heatmap of the 45 samples (Figure S1) indicates high repeatability among the replicates, confirming the reliability and validity of the experimental data.

### 2.2. Tissue-Specific DEGs Highlight Key Metabolic and Signaling Pathways

We conducted comprehensive analyses of five distinct tissue types from three cultivars. A total of 24 pairwise comparisons of differentially expressed genes (DEGs) were performed to explore the genetic landscape. In the SH2_L vs. ZS3_L comparison, 4929 genes were upregulated, while 4950 were downregulated. In SH2_L vs. ZS49_L, 5157 genes were upregulated and 3751 were downregulated. In ZS3_L vs. ZS49_L, 2750 genes were upregulated and 1772 were downregulated. A total of 1293 common DEGs were identified across the leaf tissues of the three cultivars. Notably, genes associated with the plant–pathogen interaction pathway exhibited significant differential expression. In SH2_S vs. ZS3_S, 2525 genes were upregulated and 2654 were downregulated. In SH2_S vs. ZS49_S, 3638 genes were upregulated and 3580 were downregulated. In ZS3_S vs. ZS49_S, 5538 genes were upregulated and 5545 were downregulated. A total of 1699 common DEGs were identified in stem tissues. The genes involved in the biosynthesis of secondary metabolites, flavonoid biosynthesis, and metabolic pathways showed significant differential expression.

**Figure 2:**
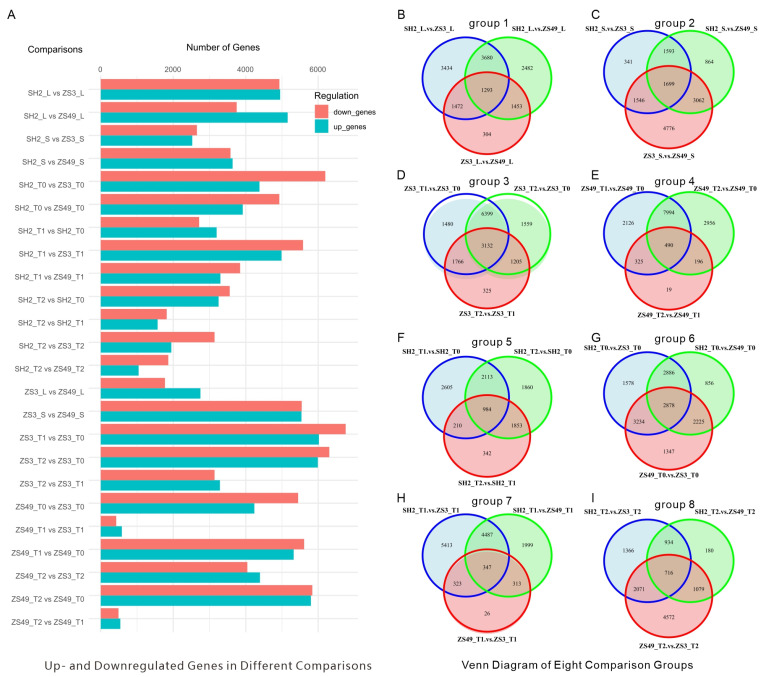
Comparisons of differentially expressed genes in various tissues of three potato cultivars. (**A**) Numbers of up- and downregulated genes in five tissues of the three cultivars. (**B**–**I**) Eight comparative analyses examining gene expression differences across tissues (leaves, stems, T0, T1, and T2) and between cultivars (SH2, ZS3, and ZS49) at different developmental stages.

A total of 3132 and 984 common DEGs were found in different tuber stages of the ZS3 and SH2 cultivars, respectively, while only 490 were detected in ZS49 (Figure 2C–E). At the T0 stage, the three varieties shared the highest number of common DEGs (2878) (Figure 2G), whereas at T1 (Figure 2H) and T2 (Figure 2I), the numbers were lower (347 and 716, respectively). Genes associated with multiple pathways, including secondary metabolite biosynthesis, phenylpropanoid biosynthesis, linoleic acid metabolism, metabolic pathways, ABC transporters, and the MAPK signaling pathway, exhibited significant differential expression (Figure S2).

### 2.3. Tissue-Specific Expression Patterns of Key Genes Involved in Regulating Potato Tuber Initiation

**Figure 3:**
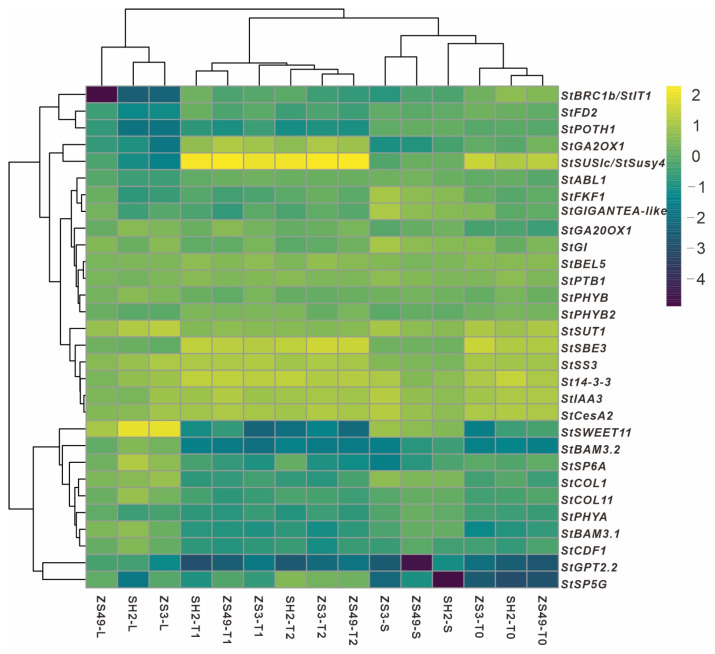
Expression patterns of key genes involved in tuber formation and development across various tissues. This figure illustrates the expression patterns of key genes in the leaves, stems, stolons, and tubers of three potato cultivars. Yellow represents high gene expression, while dark green indicates low expression. Gene expression levels were measured in FPKM (fragments per kilobase of exon model per million mapped fragments), which were logarithmically transformed, normalized, and standardized before visualization using a heatmap.

Previous studies have preliminarily revealed that photoperiod, hormones, and sugar metabolism significantly influence potato tuber formation. Based on this, 30 relevant genes were selected for expression pattern analysis. The results show that genes such as *StSWEET11*, *StSP6A*, *CYCLING DOF FACTOR 1* (*StCDF1*), and *StSP5G* had relatively high expression levels in leaf tissues, significantly exceeding those in the stem, T0, T1, and T2 tissues (Figure 3). Among the different cultivars, the expression levels of the *StSWEET11* gene were significantly higher in the ZS3-L and SH2-L samples than in the ZS49-L sample (Figure 3).

Regarding potato homologs of the *Flowering Locus T* gene, the *StSP6A*, *StCDF1*, and *StSP5G* expression levels were significantly higher in the SH2-L sample than in the ZS3-L and ZS49-L samples (Figure 3). It is well established that *StSP5G* negatively regulates *StSP6A*. In this study, *StSP5G* expression in all SH2 tissue samples was significantly lower than in the corresponding ZS3 and ZS49 tissue samples.

Genes such as the sucrose synthase genes *StSUSIc*/*StSuSy4*, the gibberellin 2-oxidase 1 gene *StGA2OX1*, and *StSBE3* (Starch Branching Enzyme 3) exhibited higher expression in tuber tissues than in leaf tissues (Figure 3). Conversely, genes such as the sucrose transporter gene *StSUT1* and the plant circadian rhythm genes *StGI* and *StGI*-like showed elevated expression in stem tissues compared with other tissues (Figure 3). The blue light receptor protein StFKF1 in potato can form a complex with GI, a core regulator of the biological clock. This study revealed that *StFKF1* and *StGI-like* were significantly upregulated in stem tissues (Figure 3). Another key factor is the zinc finger DOF family protein StCDF1, which, under inductive conditions, can indirectly activate *StSP6A* by transcriptionally repressing *CONSTANS-like1* (*StCOL1*). In this study, the significantly high expression of these genes in leaf tissues strongly (Figure 3).

Interestingly, the *StBRC1b*/*StIT1* gene exhibited extremely low expression in leaf tissues (Figure 3). However, it showed relatively high expression in the T0, T1, and T2 samples of the SH2 cultivar, surpassing the corresponding samples of the ZS3 and ZS49 cultivars (Figure 3). Additionally, this study identified that *StBAM3.1* (chloroplast β-amylase) and *StBAM3.2* exhibited relatively high expression in leaf tissues (Figure 3), while their expression was notably lower in stem and tuber tissues. A random subset of the genes mentioned above was selected for qRT-PCR validation, and the results demonstrated that all qRT-PCR outcomes were consistent with the RNA-seq transcriptome results (Figure S3).

### 2.4. Weighted Gene Co-Expression Network Analysis Identifies Tissue-Specific Modules in Potato

**Figure 4:**
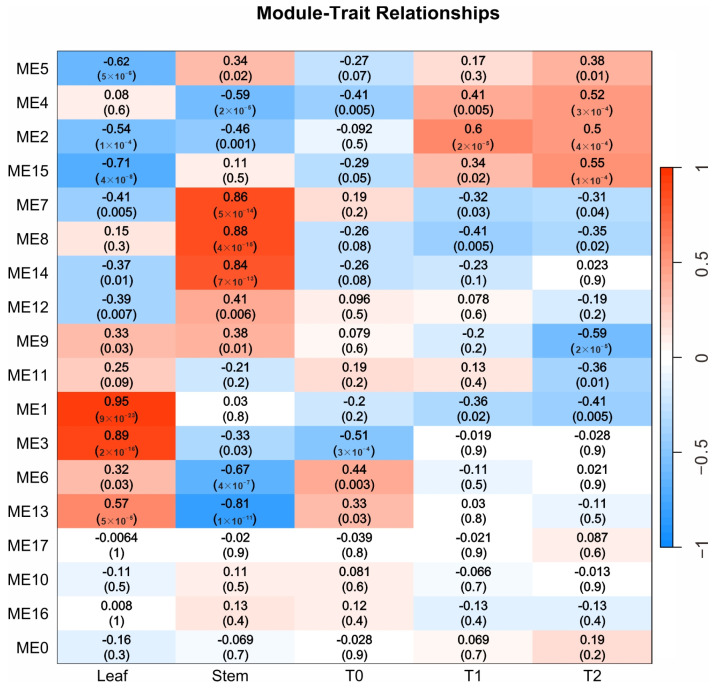
WGCNA of genes with fragments per kilobase of exon model per million mapped fragments (FPKM > 1) in five tissues of three potato cultivars, and Pearson correlation analysis between modules and tissues. The heatmap illustrates the gene expression patterns across different modules and tissues. Red indicates a positive correlation, while blue represents a negative correlation.

Weighted gene co-expression network analysis (WGCNA) is widely used to identify modules highly correlated with specific traits and to screen core genes. In this study, WGCNA was performed on three distinct potatoes cultivars using a gene set with FPKM values >1 across five tissue types. A total of 18 co-expression modules were identified (Figure 4). The ME1 (4292 genes), ME3 (1915 genes), and ME13 (215 genes) modules showed the strongest correlations with leaf tissues, with correlation coefficients of 0.95, 0.89, and 0.57, respectively. The ME2 (3516 genes), ME4 (1209 genes), and ME15 (191 genes) modules were most strongly correlated with tissues at the T1 and T2 stages, with correlation coefficients of 0.50, 0.52, and 0.55, respectively. The ME7 (550 genes), ME8 (493 genes), and ME14 (196 genes) modules exhibited the highest correlations with stem tissues, with correlation coefficients of 0.86, 0.88, and 0.84, respectively. The module most strongly correlated with the T0 stage was ME6 (713 genes), with a correlation coefficient of 0.44.

### 2.5. Co-Expression Network Reveals the Key Role of Sugar Transport and Metabolism in Conjunction with Tuberization Signaling-Associated Genes from Leaves and Stems to Tubers During Tuber Initiation

**Figure 5:**
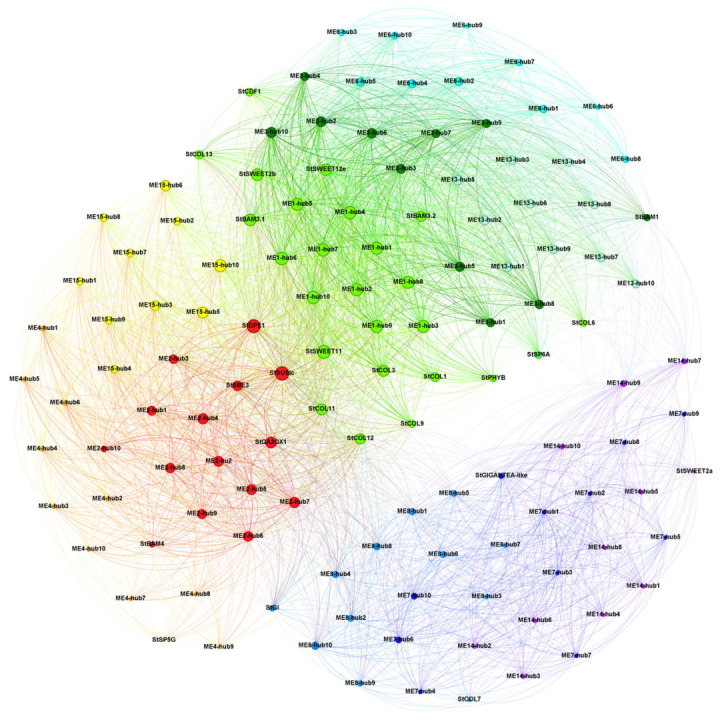
The co-expression network of core and related genes in modules associated with leaves, stems, and tubers. Different colors represent different modules, highlighting the top ten core genes within each module and genes associated with tuberization signals. The ME1, ME3, ME6, and ME13 modules, which are positively correlated with leaves, are shown in green, dark green, cyan, and light cyan, respectively. The ME7, ME8, and ME14 modules, which are positively correlated with stems, are represented in dark blue, light blue, and purple, respectively. The ME2, ME4, and ME15 modules, which are positively correlated with the T1 and T2 tuber stages, are shown in red, orange, and yellow, respectively. The lines represent edges in the network, and the color is a blend of the source node and target node colors.

By comprehensively analyzing gene correlation and connectivity within the modules, the top ten ranked genes were identified as hub genes. Based on these hub genes and reported tuberization-related genes, a co-expression regulatory network was constructed (Figure 5). The ME1, ME3, ME6, and ME13 modules were positively correlated with leaves and included *StSP6A*, *StCDF1*, *StPHYB*, *StSWEET11*, *StBAM3.1*, *StBAM3.2*, *StCOL1*, *StCOL3*, *StCOL9*, *StCOL11*, and *StCOL12*. The ME2, ME4, and ME15 modules were positively correlated with the T1 and T2 tuber stages and included *StSUSIc*/*StSuSy4*, *StGA2OX1*, *StSBE3*, and *StDPE1* (disproportionating enzyme 1). Notably, *StSP5G* was the hub gene of module ME4. The ME7, ME8, and ME14 modules were positively correlated with stem tissues and included *StGI*, *StGIGANTEA-like*, *StSWEET2a*, and *StCOL7*.

CONSTANS (CO) promotes flowering and shares structural and functional similarities with the *CONSTANS-LIKE* gene family. Members of the *COL* gene family interact with *PHYB*, influencing plant growth and development, with both regulated by the photoperiod. In this study, *StGI* and *StGIGANTEA-like* were found to connect the stem and tuber modules, while genes from the *StSWEET11*, *StSUSIc*/*StSuSy4*, and *CONSTANS-LIKE* families linked the leaf and tuber modules (Figure 5). *StSWEET11* transports sugars, *StSUSIc*/*StSuSy4* decomposes sucrose, and *StDPE1* contributes to starch metabolism. These three genes may be co-expressed in source and sink organs, coordinately regulating carbon allocation and energy metabolism.

## 3. Discussion

Potatoes originated in the Andes mountains of South America. In diploid wild-type potatoes, tuber formation strictly depends on short-day conditions and does not occur under long-day conditions. In contrast, tuberization in tetraploid cultivated potatoes is accelerated under short-day conditions but can also occur under long-day conditions [2]. Among the three tetraploid cultivated potato cultivars examined in this study, Shishu2 forms tubers the earliest (Figure 1). PHYTOCHROME B (PHYB), a major photoreceptor, perceives red and far-red light signals and plays a key role in light signal transduction and the regulation of plant growth and development. It is a key regulator of photoperiod-dependent tuberization, strongly expressed in leaves under inductive conditions and transported via the phloem to stolons to induce tuberization. The *phytochrome F* gene also regulates the development of potato leaves and stolons [9]. In this study, the expression level of *StPHYB* was highest in SH2-L, lower in ZS49-L, and lowest in ZS3-L, as confirmed by qPCR.

StSP6A is synthesized in the leaves and transported to the stolon apex via the sieve tube elements of the phloem. It interacts with StSP5G and St14-3-3 proteins to form a transcription complex [12], which regulates the initial development of tubers. In this study, the expression levels of *StSP6A*, *StCDF1*, and *StSP5G* in SH2 leaf samples were significantly higher than those in the ZS3 and ZS49 cultivars (Figure 3). Although *SP6A* expression was lowest in the stem tissues of all three cultivars, its levels during the T0–T2 period were higher than in the stem tissues of the respective cultivars, with the highest expression observed in SH2. Previous studies have reported a direct regulatory interaction between StSP6A and StSP5G in potatoes [12]. In this study, StSP5G was identified as a core gene of the ME5 module related to stem tissues, whereas SP6A exhibited the highest expression in leaves. The expression patterns of these genes align with their expected functions in cultivars with different maturity types. This study found that they belong to distinct modules with no significant co-expression relationship (Figure 5), possibly because the analysis was based on transcriptome gene expression levels rather than yeast two-hybrid protein interaction assays. In addition, epigenetic and post-transcriptional regulation can both influence tuber development [27].

The *CONSTANS* (*CO*) gene is a key regulator of flowering in the photoperiod pathway, and the *CONSTANS-LIKE* gene family shares similar structural and functional characteristics with *CO*. Previous studies have shown that members such as *COL1* and *COL2* in the *COL* gene family can interact with the PHYB protein within the nucleus [7,28]. Under long-day conditions, PHYB influences the stability and activity of COL through this interaction, thereby regulating the expression of downstream flowering-related genes and ultimately affecting flowering time [7]. At the transcriptional level, the expression of *COL* and *PHYB* is also jointly regulated by environmental factors such as the photoperiod [29]. Previous research has shown that under long-day conditions, tuber formation is delayed in plants overexpressing StCO, while *StCO*-silenced plants demonstrate tuber formation under both inhibitory and weakly inductive photoperiods, with no significant effect under strongly inductive short-day conditions. The StCOL1 protein activates *StSP5G*, which downregulates *StSP6A*, thereby inhibiting tuber formation [10,30]. A recent report indicated that the *CO* gene plays a central role in photoperiod perception [7]. The WGCNA network analysis in this study showed that COL1 (CONSTANS-like), a key gene in photoperiod signal regulation, and its family members (*COLs*) were expressed in leaves (Figure 3), forming the core of the transcriptional regulation network in the plant leaf tissues. They were co-expressed with genes such as *StSWEET11* in leaf-related modules (Figure 5), and they coordinately regulate the transport of photosynthetic metabolites from leaves to sink organs such as tubers.

Circadian rhythm regulatory genes play a crucial role in the early stages of potato tuber formation [3,5]. A previous study indicated that these genes act upstream of *StCO* and *StFT*, forming a complex with *StFKF1* and *StCDF1* to regulate tuber formation under photoperiodic control [2]. The WGCNA network analysis in this study further showed that *StGIGANTEA* (*StGI*) and *StGI*-*like* occupied key network nodes in the stem- and tuber-related modules (Figure 5), reinforcing their role in coordinating the gene regulatory network that governs stem and tuber development.

Sucrose not only serves as a substrate for energy production in developing tubers and starch synthesis but also acts as a crucial signal regulating tuberization. Genes such as *StSUSIc*/*StSuSy4*, *StSWEET11*, and *StBAM3.1* are co-expressed with circadian rhythm regulatory genes (*COLs*), collectively modulating the flow of photosynthetic metabolites to sink organs such as tubers. Genes involved in starch biosynthesis play a role in the early stages of potato tuber development [31]. The *SWEET11* gene is continuously expressed in vascular tissues, facilitating long-distance sugar transport [32]; the *SUS* gene is highly expressed in actively growing tissues, such as root and shoot tips [33], providing energy and carbon skeletons. The *DPE1* gene regulates short-chain maltooligosaccharide dynamics, affecting the initial stages of starch synthesis [34,35]. The *SBE3* (Starch Branching Enzyme *3*) is a key determinant of starch synthesis and structure in plant organs, which also impacts plant growth, development, and adaptation processes [36]. This study further revealed that metabolic-related genes such as *StSUSIc*/*StSuSy4*, *StDPE1*, and *StSBE3* were co-expressed in tuber-related modules such as ME2; they occupied central positions in the WGCNA network and were co-expressed with sucrose transporters such as *StWEET11*. These highly correlated co-expressions position them at the core of the regulatory network (Figure 5), underscoring the pivotal role of sucrose transport, starch synthesis, and metabolic regulatory genes in leaf and tuber formation, source–sink carbon allocation, and energy metabolism [37,38]. Additionally, many core genes are linked to primary and secondary metabolic pathways. Among these, ME13-hub9 (Soltu.DM.07G013620) encodes a starch synthase, while ME13 is a core gene in the leaf-related module. These findings highlight the value of our transcriptome data in unravelling transcriptional regulatory networks across potato tissues, including leaves and tubers.

## 4. Materials and Methods

### 4.1. Plant Materials

Tetraploid potato cultivars, including the early-maturing cultivars ‘Zhongshu 3’ (ZS3) and ‘Shishu 2’ (SH2) and the late-maturing cultivar ‘Zhongshu 49’ (ZS49), were used. On 20 May 2024, seed potatoes were planted in a container filled with peat moss and vermiculite (1:1, *v*/*v*) under net-covered greenhouse conditions at the Chabei Base of the Institute of Vegetables and Flowers, Chinese Academy of Agricultural Sciences (41°25.2′ N, 114°56.4′ E). Water and fertilizer management was carried out through a conventional drip irrigation system. The third leaf from the top and its connected stem, unswollen stolon (T0), swollen sub-apical stolon region (T1), and tissues from the initial tuber formation stage (T2, with a tuber diameter of approximately 1 cm) were collected. Sub-apical swelling of stolons (T1) was observed to begin on day 7 after seedling emergence. For the cultivars ‘Shishu 2’, ‘Zhongshu 49’, and ‘Zhongshu 3’, the time points at which T1 status was reached were approximately 11, 15, and 19 days after seedling emergence, respectively. A total of 45 samples (three biological replicates of five tissue types from three cultivars) were rapidly frozen in liquid nitrogen and stored at −80 °C until use.

### 4.2. RNA Extraction and cDNA Library Construction

Total RNA was extracted from tissues using an RNAprep Pure Plant Plus Kit (TIANGEN, Beijing, China). RNA quality was assessed using an Agilent 2100 Bioanalyzer (Agilent Technologies, Santa Clara, CA, USA). The transcriptome library was prepared with a Hieff NGS^®^ Ultima Dual-mode RNA Library Prep Kit (Premixed version) (Yeasen, Shanghai, China). Poly(A)-tailed mRNA was first enriched using Oligo(dT) magnetic beads. cDNA synthesis was performed in two sequential steps, followed by end repair and A-tailing. MGI-specific adapters (MGI Tech Co., Ltd., Shenzhen, China) were then ligated to the products. After adapter ligation, PCR amplification was conducted to generate the cDNA library. RNA libraries were sequenced on the DNBseq-T7 platform at the Smartgenomics Technology Institute (Tianjin, China).

### 4.3. RNA Sequencing and Data Analysis

Sequencing was performed on the DNBSEQ-T7 platform based on the libraries’ effective concentration and data output requirements. Quality control was conducted using fastp (Version 0.20.0) with default parameters to filter out low-quality reads. High-quality reads were then aligned to the reference genome using HISAT2 (Version 2.2.1) to determine their genomic positions on the reference genome [2]. Transcripts for each sample were assembled using StringTie (Version 1.3.3). For samples with biological replicates, differential expression analysis between two comparative groups was performed using DESeq2 (Version 1.24.0). The Benjamini–Hochberg method was applied to adjust *p*-values and control the false discovery rate. Genes with adjusted *p*-values < 0.05 identified by DESeq2 were classified as differentially expressed genes (DEGs). Significant differential expression was determined using adjusted *p*-values and |log_2_FC| > 1 as thresholds. The gene expression levels were quantified as fragments per kilobase of exon per million mapped reads (FPKM). Pathway enrichment analysis of DEGs was conducted using the Kyoto Encyclopedia of Genes and Genomes (KEGG) database, with *p*adj < 0.05 as the threshold for significant KEGG pathway enrichment.

### 4.4. Weighted Gene Co-Expression Network Analysis

Genes with an FPKM > 1 were selected for WGCNA (Version 1.73). The soft power value was set to 6, and the network type was defined as unsigned. The minimum module count was set to 100, with a merging threshold of 0.25. Subsequently, module–trait correlations were calculated. Based on intramodular connectivity (MM ≥ 0.7) and correlation (≥ 0.5), the top 10 genes within each module were identified as core genes. Modules significantly correlated with traits were selected and combined with previously reported relevant genes [25,39]. Co-expressed genes with a weight > 0.1 were filtered. Gephi 0.10.1 software was used to generate the network layout using the Fruchterman–Reingold algorithm, with node sizes ranked by average degree centrality. By integrating core gene connectivity and correlation, the top 10 core genes and differentially expressed genes of interest were selected for visualization.

### 4.5. Validation by Quantitative PCR

Eight genes from co-expression modules associated with stolons or tubers were selected for qRT-PCR validation. cDNA from five tissues (leaves, stems, T0, T1, and T2) of three cultivars (SH2, ZS3, and ZS49) was used as the template. Elongation factor-3e (*StELF3e*) served as the internal reference gene [40]. The quantitative PCR reaction system was employed, and the relative expression levels were calculated using the 2^−ΔΔCt^ method, as previously reported [20]. All qPCR primers are listed in Table S3. The housekeeping gene *ELF3e* was used as a control.

## 5. Conclusions

In conclusion, this study provides a comprehensive analysis of the gene co-expression network during the development of different potato tissues. The examination of differentially expressed genes and KEGG pathways revealed significant variations in metabolic and secondary metabolism-related pathways across cultivars and tissue types. Weighted gene co-expression network analysis identified gene modules associated with the initial formation of leaves, stems, and tubers. Among these, *StSP5G* is the core gene of the ME4 module, which is strongly linked to tubers. *StPHYB*, *StCOL1*, *StSWEET11*, and *StSP6A* are part of the leaf tissue-related module and exhibit significant co-expression relationships. *StSUSIc*/*StSuSy4* and *StGA2OX1* are in the stem tissue-related module, also showing strong co-expression relationships. *StSWEET11* and *StSUSIc*/*StSuSy4* play key roles in connecting the co-expression networks of leaf and stem tissues, while *StGI* and *StGI-like* are crucial for linking the stem and tuber networks. Therefore, this study provides detailed insights into the signal co-expression network from leaves and stems to tubers, enhancing our understanding of the transcriptional coordination among tissues during potato growth and development, as well as the signaling pathways involved in sucrose metabolism and transport.

## Data Availability

All data supporting the findings of this research are available within the paper and within the Supplementary Data published online.

## Supplementary Materials

The following supporting information can be downloaded at https://www.mdpi.com/article/10.3390/ijms26115278/s1.
