# Supplementary material for: Transcriptome Analysis Reveals Co-Expression Regulation of Sugar Transport and Signaling Networks in Initiating Stolon-to-Tuber Transition in Potato
Source: Int J Mol Sci. 2025 May 30;26(11):5278. doi: 10.3390/ijms26115278 (PMC12154728; doi:10.3390/ijms26115278)
Supplement: Supplementary file 1 [file ijms-26-05278-s001.zip › Figure S1-S3.pdf]

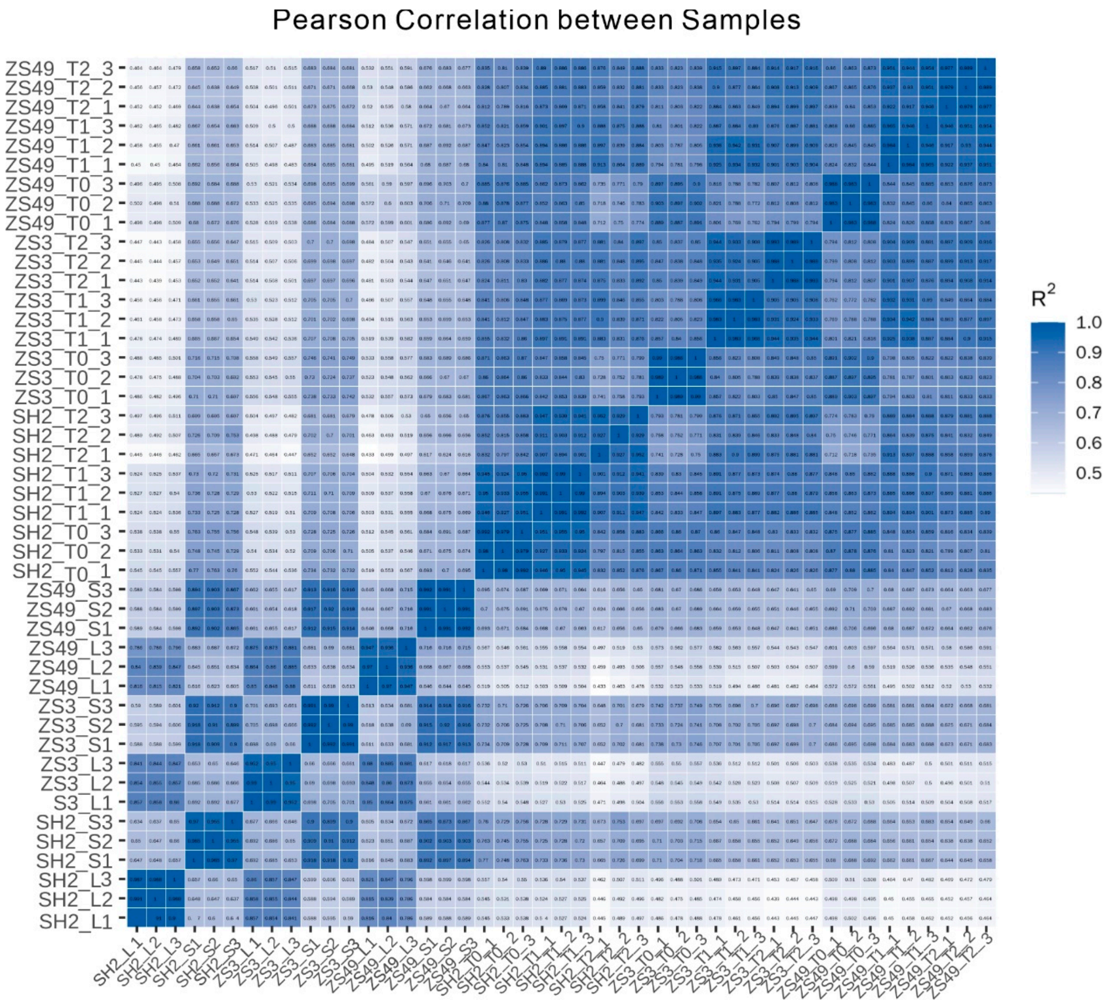

Figure S1. Heatmap of Pearson correlation among 45 samples. The horizontal and vertical coordinates in the graph represent the names of different samples. The color represents the value of the square of the correlation coefficient (R^2) between samples. The color scale on the right shows that the darker the color (with values close to 1.0). The correlation among samples within the three replicates is significantly higher than that among samples from other tissues.

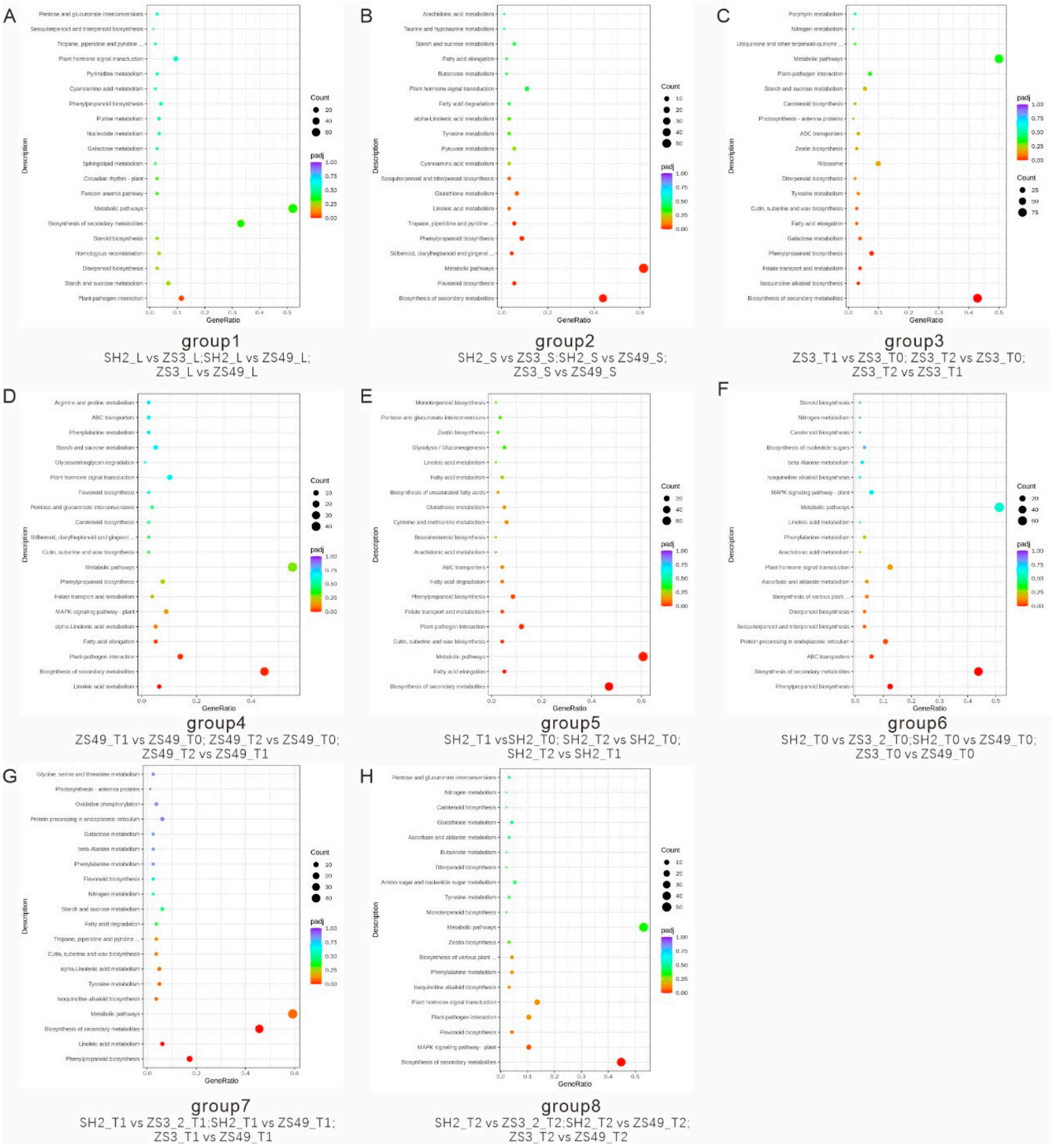

Figure S2. Comparison analysis of KEGG differential metabolic pathways among 8 groups of different varieties and tissues. Comparisons were made between leaves (A), stems (B), T0 (C), T1 (D), T2 (E) of different varieties and tubers at different stages of ZS3 (F), ZS49 (G), and SH2 (G). KEGG enrichment analysis was carried out on the differential metabolic pathways. The top 20 KEGG pathways with the most significant differences were selected for graphing. The horizontal axis represents the ratio of the number of differential genes annotated to KEGG pathways to the total number of differential genes. The vertical axis represents the KEGG pathways. The size of the dots indicates the number of genes annotated to the corresponding KEGG pathway, and the color gradient from red to purple represents the decreasing degree of enrichment significance.

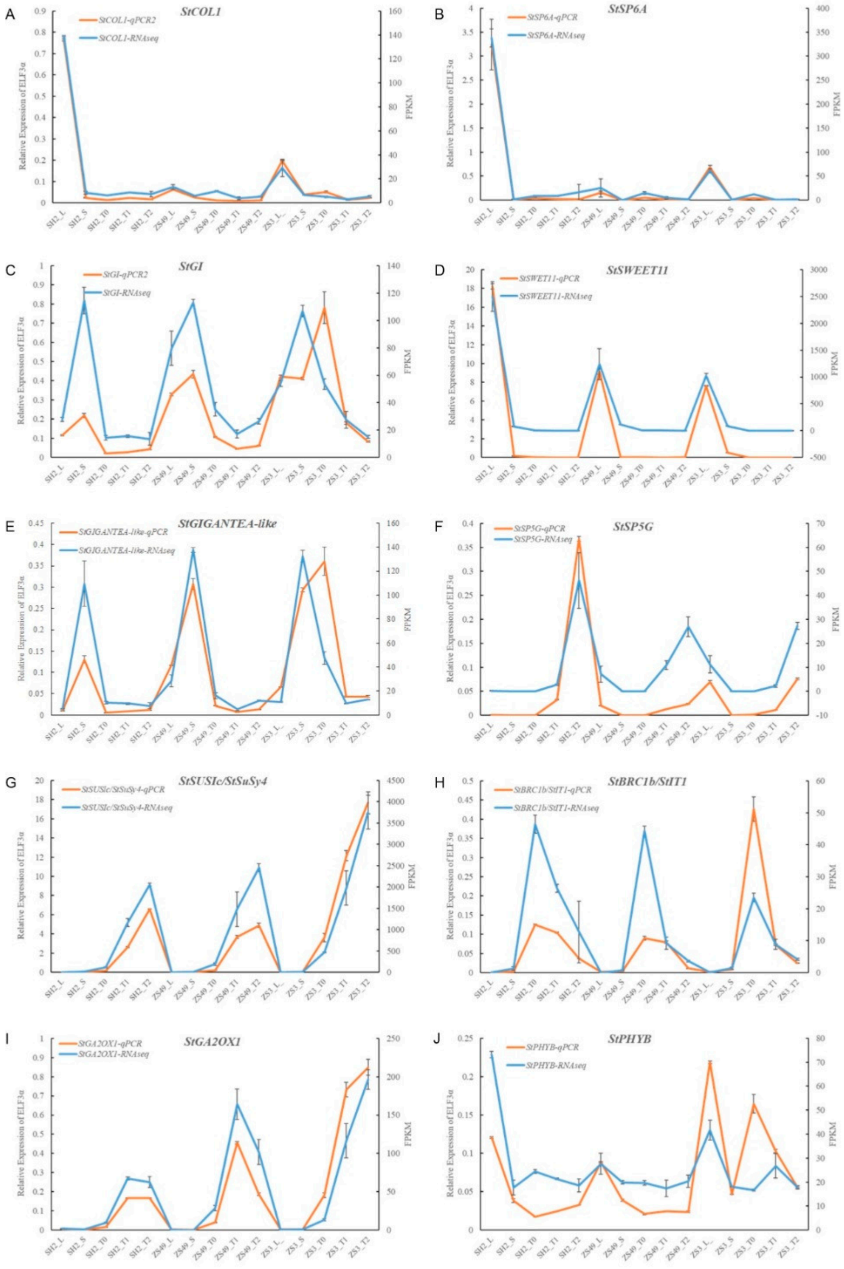

Figure S3. qPCR verification of tuberization signaling and metabolic genes in the co-expression network. *StCOL1*(A)、*StSP6A*(B)、*StGI*(C)、*StSWEET11*(D)、*StGIGANTEA-like*(E), *StSP5G*(F), *StSUSIc/SuSy4*(G), *StBRC1b/StIT1*(H),*StGA2OX1*(I), and StPHYB (J) were selected for qPCR verification. Orange represents the values from qPCR, and blue represents the FPKM values from RNA-seq.
