# Supplementary figures and images for: Transcriptome Analysis Reveals Co-Expression Regulation of Sugar Transport and Signaling Networks in Initiating Stolon-to-Tuber Transition in Potato
Source: Int J Mol Sci. 2025 May 30;26(11):5278. doi: 10.3390/ijms26115278 (PMC12154728; doi:10.3390/ijms26115278)

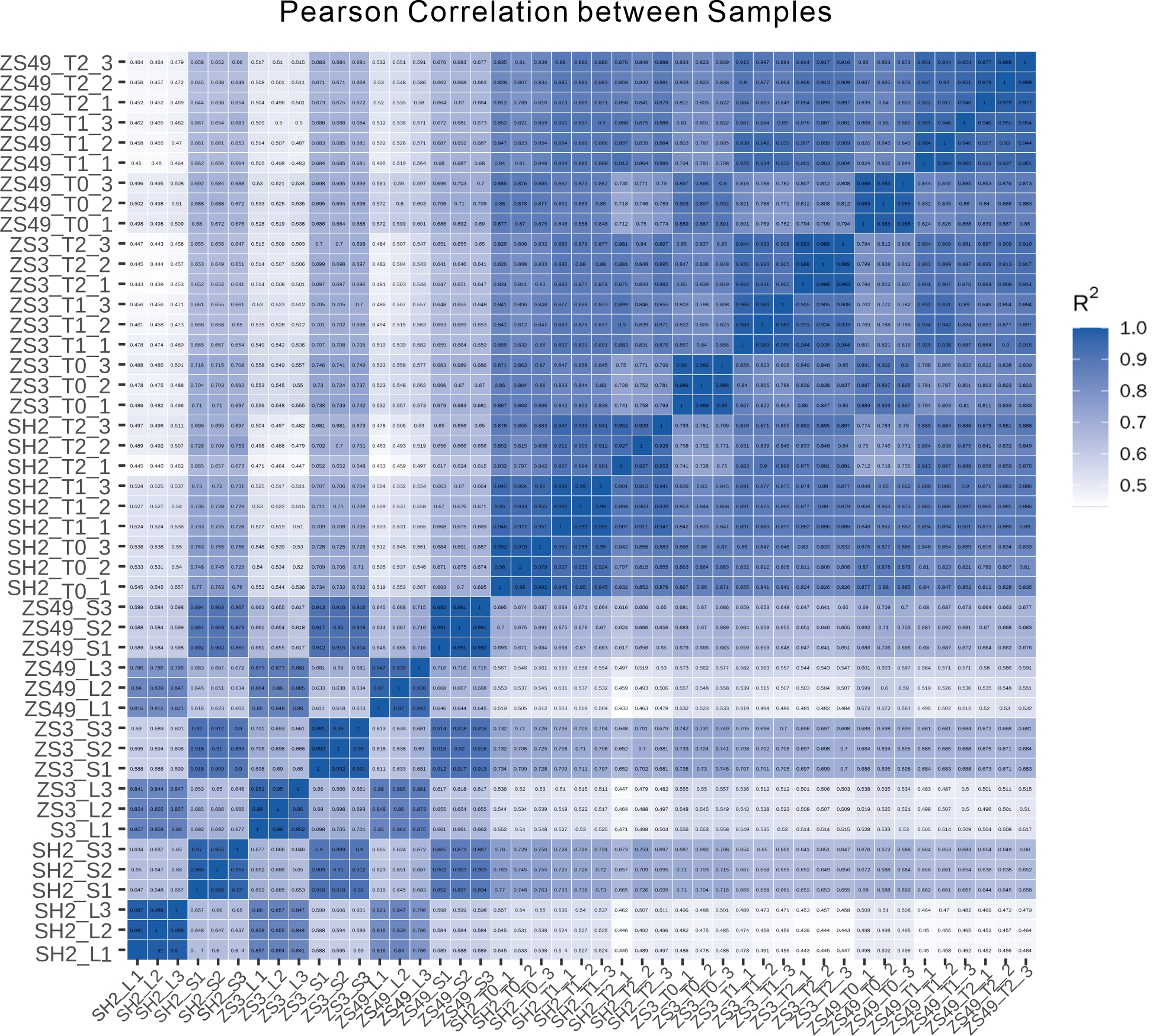

Supplement: Supplementary file 1 [file ijms-26-05278-s001.zip › Figure S1-0312.jpg]

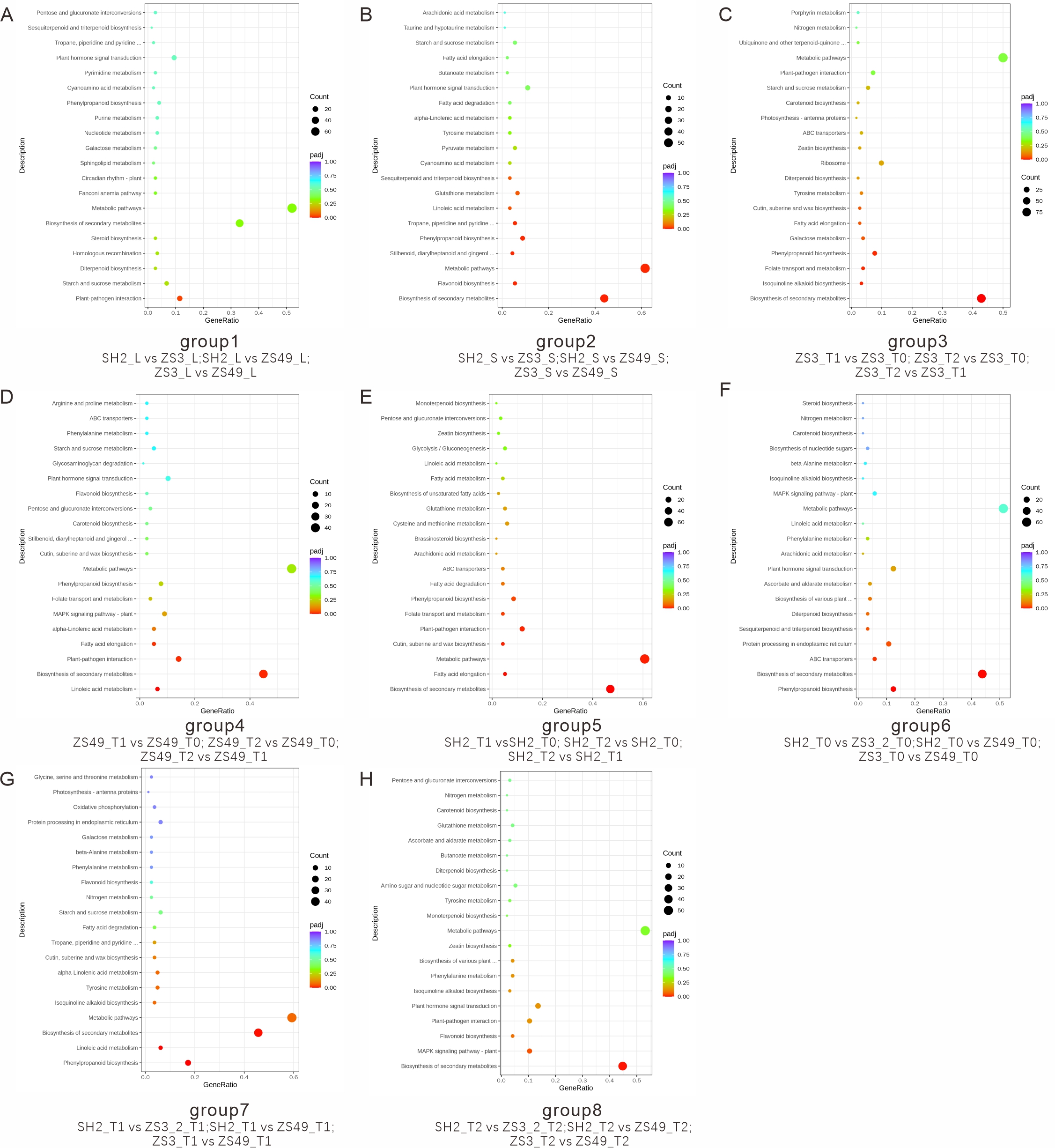

Supplement: Supplementary file 1 [file ijms-26-05278-s001.zip › Figures S2.jpg]

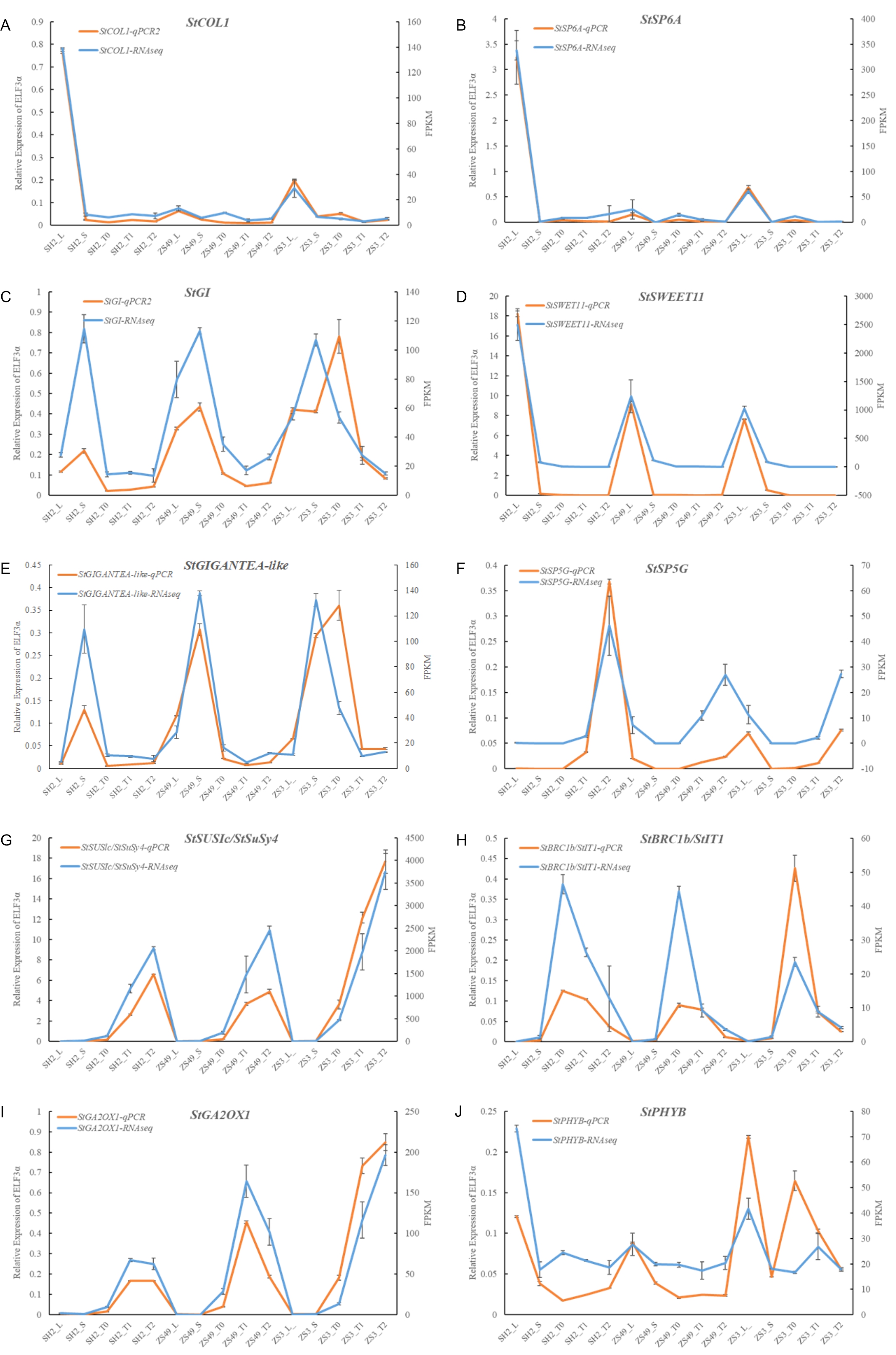

Supplement: Supplementary file 1 [file ijms-26-05278-s001.zip › Figures S3.jpg]
